# Motion correction of simultaneous brain PET/MR images based on tracer uptake characteristics

**DOI:** 10.1186/s40658-025-00789-6

**Published:** 2025-07-30

**Authors:** Sheng-Chieh Chiu, Jose Angelo U. Perucho, Yu-Hua Dean Fang

**Affiliations:** 1 Department of Biomedical Engineering, School of Engineering, University of Alabama at Birmingham, Birmingham, AL USA; 2 Department of Radiology, Heersink School of Medicine, University of Alabama at Birmingham, 1720 2nd Ave S, Birmingham, AL 35294 USA; 3 Department of Neurology, Heersink School of Medicine, University of Alabama at Birmingham, Birmingham, AL USA; 4 Alzheimer’s Disease Research Center, Heersink School of Medicine, University of Alabama at Birmingham, Birmingham, AL USA

**Keywords:** Simultaneous PET/MR, Motion correction, Amyloid imaging, PET quantification

## Abstract

**Background:**

Simultaneous PET/MR imaging enables precise anatomical localization and PET quantification by reducing PET-to-MR misalignments. However, involuntary motion during scans may still cause misalignment and quantification imprecision. Current mutual information (MI)-based co-registration methods do not account for the tissue-specific uptake patterns of PET and therefore could result in suboptimal alignment. To address this, we proposed a novel image co-registration method, namely the tracer characteristic-based co-registration (TCBC) method, which takes advantage of specific PET uptake patterns within a selected anatomical region to improve the image alignment and PET quantification.

**Results:**

TCBC was evaluated using simulation and in vivo ^18^F-Florbetapir PET/MR data from the OASIS-3 dataset. In simulations, TCBC demonstrated superior alignment accuracy with lower root mean square error and higher R-squared values compared to the conventional MI-based co-registration from FreeSurfer in recovering the simulated patient motion. In the retrospective human study, we evaluated the detectability of age-related amyloid burden in healthy controls under different co-registration methods as a demonstrative use case. TCBC significantly enhanced the detectability of age-related amyloid burden with stronger correlations across all five regions of evaluation, such as the medial orbitofrontal cortex (*p* < 0.001), precuneus (*p* = 0.004), and early amyloid-β composite (*p* = 0.002), compared to FSMC (*p* = 0.004, 0.007, and 0.006, respectively) and uncorrected (*p* = 0.378, 0.023, and 0.039, respectively) methods. Bootstrap analyses also confirmed TCBC’s robustness in smaller samples, yielding tighter confidence intervals and lower means of p-values, such as 0.032 (95% CI: 0.029–0.035) in the precuneus and 0.008 (CI: 0.007–0.010) in the medial orbitofrontal cortex, outperforming FSMC (*p* = 0.046 with CI: 0.042–0.049, and *p* = 0.040 with CI: 0.036–0.044, respectively).

**Conclusions:**

The TCBC method reduces image misalignment, improves PET quantification, and may have a good potential for being applied to both research and clinical studies with simultaneous brain PET/MR.

**Clinical trial number:**

Not applicable.

**Supplementary Information:**

The online version contains supplementary material available at 10.1186/s40658-025-00789-6.

## Background

Simultaneous Positron Emission Tomography and Magnetic Resonance (PET/MR) scans allow PET and MR studies to be acquired during the same session and have been widely used in clinical and research settings. Anatomical MR is often used to generate brain parcellation and segmentations for quantitative analysis of PET. One of the main advantages of simultaneous PET/MR is a marked reduction of the misalignment between the two modalities compared to separate PET and MR sessions. However, depending on the scanning protocols of PET/MR studies, misalignment due to the involuntary subject motion may still occur especially when there is a considerable time difference between the PET and MR acquisitions. Since PET typically requires a longer acquisition time (~ 20–30 min static and 60–90 min dynamic [1]) than anatomical MR (~ 4–8 min), involuntary motion of the subject during the scan could lead to misalignment between the two modalities and an imprecise quantification of PET data with MR-based segmentation [2–5]. Involuntary motion can be particularly pronounced in patients suffering from neurodegenerative diseases such as cognitive impairment or movement disorders [6, 7]. In integrated PET/MR scanners, even small patient motion can cause noticeable image degradation and quantification inaccuracies [5, 8, 9]. Such misalignment between PET and MR images shall be corrected to reduce the measurement error of the PET uptake made under the MR-based segmentation.

Image co-registration methods have been developed for correcting the PET-to-MR misalignment and those based on the mutual information (MI) theory [10] have become popular among current approaches. MI-based registration seeks to maximize the statistical dependence between two sets of imaging signals for multi-modal image co-registration [11–13]. However, MI-based registration considers each voxel intensity value on the same modality independently when calculating statistical dependence and assumes no spatial co-dependence between voxels. This approach does not account for the patterns of voxel intensity in different tissues. For example, substantial tracer uptake due to non-specific binding is commonly observed in the cerebral white matter (WM) in amyloid PET, a main imaging tool for Alzheimer’s Disease (AD) evaluation [14]. Such knowledge of well-established tracers and their uptake patterns may be helpful to guide the PET-to-MR registration, but MI-based registration does not make use of this a priori information as only the MI between PET and MR intensities on each individual voxel is considered. As a result, MI-based registration methods may not yield an optimal solution when registering PET and MR images [13, 15]. Woo et al. have shown that different patterns of voxel intensity between the two images being registered can negatively impact the performance of conventional MI-based co-registration methods [16]. This is attributed to the fact that such variations can skew the joint histogram, which is pivotal for calculating MI, leading to inaccurate co-registration even when the images to be aligned are structurally identical. In the context of brain PET/MR imaging, T1-weighted (T1w) anatomical MR images distinguish various brain regions by T1 relaxation times, while the intensity in PET images is primarily determined by the molecular or metabolic characteristics of the tissue. The differences in the underlying physical basis of PET and MR signals do not necessarily guarantee that the optimal solution of the objective function with MI falls at the optimal co-registration.

Recognizing this challenge, we were motivated to propose a new PET-to-MR co-registration method that reduces the impact of signal discrepancies during the co-registration. Instead of relying on MR intensities, we utilize MR-derived anatomical segmentation to guide image co-registration for this novel method, named tracer characteristic-based co-registration (TCBC), which leverages the tracer uptake pattern within a selected target ROI. We compared the proposed method to the state-of-the-art FreeSurfer *mri_coreg* (FSMC) function [17], which employs normalized MI [18] for co-registration. The performance of TCBC was evaluated using simulation and human amyloid PET/MR data from the Open Access Series of Imaging Studies-3 (OASIS-3) dataset [19].

## Methods

### Tracer characteristic-based co-registration

Conceptually, TCBC registers MR to PET or PET to MR by optimizing the mean PET uptake measured from a pre-selected target ROI that has a well-documented radiotracer uptake characteristic. The optimization can be either a maximization or minimization process depending on whether the selected target ROI is known for a high or low tracer uptake. Moreover, the selected target ROI shall pose an intensity contrast with the adjacent tissues. We hypothesize that, when there is a known and significantly strong PET contrast between the selected target ROI and its surrounding tissue, the ideal co-registration of the selected target ROI and the PET images shall lead to an optimally measured mean PET intensity within this target ROI. For example, if a given brain structure has a uniformly high PET uptake while the surrounding tissue has significantly reduced uptake, the ROI of such a structure will yield the maximal measurement of the mean PET intensity when the ROI is ideally co-registered with PET.

We chose amyloid PET scans to demonstrate the feasibility of TCBC in this study. Cerebral WM was identified as a good candidate to serve as the selected target ROI, as WM typically shows a high and uniform non-specific binding of Aβ tracers such as ^18^F-Florbetapir (^18^F-FBP) in both cognitive normal (CN) subjects and patients with cognitive impairment such as AD [20]. There is also a very strong signal contrast between WM and brain ventricles which typically do not retain amyloid tracers. This WM-to-ventricle contrast will be helpful in guiding the co-registration in patients with high amyloid burdens in gray matter (GM) that could lead to blurred boundaries between GM and WM on PET [21]. These consistent contrast patterns between GM, WM and ventricles enable the use of this method in healthy subjects or patients.

To implement TCBC for PET-to-MR co-registration, we first assume that there is no distortion or strong artifacts in PET and MR images and the co-registration can be achieved through a rigid body transformation without scaling, reflection, or shearing. TCBC is then designed to optimize the affine transformation matrix *M* with six degrees of freedom determined by translation (*t*_*x*_, *t*_*y*_, *t*_*z*_) and rotation (*θ*_*x*_, *θ*_*y*_, *θ*_*z*_) along the three axes, as shown in Eq. 1:

1 $$\begin{array}{l}M\,({t_x},\,{t_y},\,{t_z},\,{\theta _x},\,{\theta _y},{\theta _z})\,\\= \,T\,({t_x},\,{t_y},\,{t_z})\,{R_Z}\,({\theta _Z})\,{R_y}\,({\theta _y})\,{R_X}\,({\theta _x})\end{array}$$

where *T* (*t*_*x*_, *t*_*y*_, *t*_*z*_) denotes translation by amount *t*_*x*_, *t*_*y*_, and *t*_*z*_ in the x, y, and z axes, respectively. *R*_*X*_ (*θ*_*x*_), *R*_*Y*_ (*θ*_*y*_), and *R*_*Z*_ (*θ*_*z*_) are the rotations within the y-z plane, x-z plane, and x-y plane by *θ*_*x*_, *θ*_*y*_, and *θ*_*z*_, respectively. These transformation parameters serve as the decision variables in the optimization algorithm. The mean intensity function can be expressed as Eq. 2:

2 $$\begin{array}{l}{I_{mean\,}}({t_x},{\rm{ }}{t_y},{\rm{ }}{t_z},{\rm{ }}{\theta _x},{\rm{ }}{\theta _y},{\rm{ }}{\theta _z})\\= \,\,\sum \,((M\,({t_x},{\rm{ }}{t_y},{\rm{ }}{t_z},{\rm{ }}{\theta _x},{\rm{ }}{\theta _y},{\rm{ }}{\theta _z})\,S) \otimes \,V)\,/N\end{array}$$

where *I*_*mean*_ (*t*_*x*_, *t*_*y*_, *t*_*z*_, *θ*_*x*_, *θ*_*y*_, *θ*_*z*_) is mean intensity within the selected target ROI after its spatial transformation. *S* and *V* denote selected target ROI as a binary mask and the PET image volume, respectively. *M* (*t*_*x*_, *t*_*y*_, *t*_*z*_, *θ*_*x*_, *θ*_*y*_, *θ*_*z*_) is the affine transformation matrix defined above and applied to the target ROI *S*. ⊗ denotes the element-wise convolution operation to calculate intensity values resulting from the transformed ROI and the PET volume. *N* is the number of voxels in the target ROI after transformation. The objective function to minimize through numerical optimization was then formulated as Eq. 3:

3 $$\mathop {\min \,}\limits_{{t_{x,\,}}{t_{y,\,}}{t_{z,\,\,{\theta _x}\,,\,{\theta _y}\,,\,\theta z\,,}}} \{ - {I_{mean}}\,({t_x},\,{t_y},{t_z},\,{\theta _x},\,{\theta _y},{\theta _z})\} $$

The TCBC method technically is not requiring any specific optimizer. In this study, we used the *fminsearch* function in MATLAB R2023b based on the Nelder-Mead simplex algorithm as the optimizer for the testing and the feasibility evaluation [22]. In practice, such optimization may often be affected by the choice of initial guesses. To reduce the bias in parameter optimization due to the choice of initial guesses, 100 sets of initial guesses were randomly generated for each numerical optimization process with the specified ranges of [-10 mm, 10 mm] for translation and [-10°, 10°] for rotation. These parameters sets were used by TCBC as the initial entries to find the ideal transformation matrix. *fminsearch* is an unconstrained optimization routine, so there were no upper or lower bounds for searching the six transformation parameters. The set of transformation parameters that generated the lowest output of the objective function was selected to compose the optimized transformation matrix. This matrix was then directly applied to both the T1w MR image and the corresponding MR-derived segmentation masks, aligning them individually with the PET image to complete the co-registration.

### Imaging dataset

Human imaging data from the OASIS-3 dataset were used to conduct both a simulation and a retrospective human study for evaluating the TCBC performance. The OASIS-3 study was a research initiative led by the Alzheimer’s Disease Research Center (ADRC) at Washington University, St. Louis and involved data from both healthy controls and patients with cognitive impairment. The dementia status of the participants was determined with the Clinical Dementia Rating Scale (CDR) [23]. For participants with available ^18^F-FBP PET/MR data, those who meet the following inclusion criteria were included in this study: (1) Participants with zero CDR scores over all available longitudinal OASIS-3 evaluations, indicating no clinical cognitive impairments during their study participation. (2) Participants that went through the full dynamic PET/MR acquisition. (3) The time interval between the participant’s PET/MR scan and the corresponding ADRC clinical assessment was less than 180 days. Key clinical characteristics such as the participant’s age at scan, gender, and apolipoprotein E (APOE) genotypes were also collected.

### Image acquisition and processing

Simultaneous PET/MR imaging was performed using a Siemens BioGraph mMR scanner. Aβ deposition was measured using ^18^F-FBP [24]. Participants received an intravenous bolus of 10 mCi of ^18^F-FBP, followed by 70 min of dynamic PET/MR imaging, which included 4 ⨯ 15 s frames, 8 ⨯ 30 s frames, 9 ⨯ 1 min, 2 ⨯ 3 min frames, and 10 ⨯ 5 min frames. For a few participants who were unable to tolerate the full dynamic scan, PET/MR scans began 50 min after bolus injection with a dynamic 20 min (4 × 5 min frames) PET acquisition. The acquired PET data were reconstructed using an Ordinary Poisson Ordered Subsets Expectation Maximization (OP-OSEM) algorithm. Structural T1w imaging was performed at the beginning of the dynamic PET acquisition with an approximate duration of 4 to 5 min. Volumetric parcellation was generated using FreeSurfer 7.3.2 for each T1w image [25].

### Study design

We conducted two studies, one with simulated data and another with in vivo human data, to evaluate the performance of TCBC in comparison to FSMC. In the simulation study, we created artificial motion to the MR data and tested if the added motion can be properly corrected by TCBC. Furthermore, we evaluated whether TCBC will provide an improved motion correction when compared to FSMC. To generate data with simulated motion, we selected a participant from the OASIS-3 dataset who only underwent a 20-min PET/MR scan at the 50th minute post injection, instead of the full dynamic scan. In this selected participant, we used the 50–55 min ^18^F-FBP PET frame as the T1w MR was acquired simultaneously during this frame for this session. This scan hence provides well-registered PET and MR data because of the minimal time difference between the selected ^18^F-FBP PET frame and the T1w scan. We assumed that this minimal time difference would prevent the occurrence of substantial motion between the T1w and PET acquisitions. Visual evaluation also confirmed that there was no noticeable misalignment.

For the simulation study, we simulated noisy PET images with artificial motion to evaluate the performance of TCBC and FSMC in recovering the simulated motion. The simulation process involved several steps. First, the PET image from the selected participant was forward-projected into 2D sinograms on a slice-by-slice basis using ‘*radon*’ function in MATLAB. Second, the resulting sinograms were scaled to estimated counts that would be expected from an ^18^F-FBP scan, based on the injected dose of 370 MBq, an approximated brain uptake of 5% total injected dose [26], a given acquisition duration and the system-level counting sensitivity of 15 kcps/MBq for Siemens Biograph mMR [27]. Third, Poisson noise was added to the scaled sinogram. Finally, the noisy sinograms were reconstructed into images using MATLAB’s iradon function of Filtered Back-Projection (FBP) with a Hamming filter. We have simulated two noise levels under acquisition time of 5 and 20 min to evaluate the impact of noise for motion correction. For both conditions, we randomly generated 500 sets of simulated motion in the form of affine transformation matrices, each comprising randomly generated translation and rotation parameters within the ranges of [-10 mm, 10 mm] for translation and [-10°, 10°] for rotation as the ground truth of the simulated motion. Each matrix was applied to the T1w MR scan and MR-derived segmentation of the selected participant to generate simulated PET/MR pairs with artificial misalignments. TCBC and FSMC were then applied individually and independently for PET/MR co-registration. For each method, the root mean square error (RMSE) values were calculated to quantify the differences between the optimized co-registration parameters and the ground truth. Additionally, R-squared values were derived from linear regression to examine the consistency between the estimated parameters and the ground truth.

In the study with in vivo human imaging data, we designed a retrospective study to evaluate how the proposed co-registration method will affect the detectability of the association between PET measurements and physiological or metabolic conditions under investigation. We chose the well-known association of age and PET-measured amyloid burden in older adults as the target endpoint to evaluate the performance of different co-registration strategies. Prior reports have shown that amyloid burden increases with age in cognitively normal older individuals as part of the normal aging process [28–30]. It can be expected that, when there is a misalignment between PET and MR, the MR-based segmentation adds additional and undesired variance to the PET measurements, reduces the PET quantification precision, and degrades the detectability of age-dependent increase of amyloid standardized uptake value ratio (SUVR) in the given cohort. As a result, when an accurate co-registration is applied to PET/MR data with potential misalignment, precision of SUVR measurement can be restored and detectability of correlation between age and amyloid burden shall be improved. Therefore, we used the detectability of age-amyloid association as an indirect measurement of the image co-registration performance.

This retrospective study was performed with CN subjects and their SUVR across several brain ROIs. Participants aged 60 to 80 who maintained a CDR of 0 on all available ADRC clinical assessments constituted this cohort. For the TCBC method, the last PET frame from each scan was used for co-registration. This frame was chosen since Wong et al. has reported that the contrast between the GM and WM reaches the maximum at approximately 70 min for a typical dynamic ^18^F-FBP scan in both AD and CN participants [20], thereby providing the sharpest anatomical boundaries for image co-registration. Five target ROIs were selected due to their established age-amyloid association demonstrated in the literature, including precuneus, anterior cingulate, posterior cingulate, medial orbitofrontal, and an early Aβ composite comprising the aforementioned ROIs [31, 32]. ^18^F-FBP SUVR values were measured from the summation of the last four PET frames from the dynamic study (50 to 70 min post-injection) as it is the most common time window for amyloid SUVR computation [33–35]. An eroded WM ROI was used as the reference region for SUVR calculation [36–38]. WM erosion was done in accordance with the guideline proposed in Alzheimer’s Disease Neuroimaging Initiative (https://adni.loni.usc.edu/) to reduce spillover from surrounding regions. We used linear mixed-effect models (LMMs) with fixed-effect variables of age, sex, education, and APOE ε4 carriers, to determine their correlation with the ^18^F-FBP SUVR in target ROIs. We specifically focused on the age-SUVR association in this study.

We also designed a task under smaller sample sizes within the original cohort with the bootstrapping technique to evaluate the statistical power of TCBC-based SUVR compared to those measured under FSMC and uncorrected data. We performed bootstrap sampling and repeated the LMM analysis on each of the bootstrap samples [39]. Specifically, we generated 1000 bootstrap sets, each containing 100 subjects randomly drawn from the originally eligible CN subjects. For each bootstrap sample, we calculated ^18^F-FBP SUVR under three co-registration conditions (uncorrected, FSMC, TCBC). We then fitted the SUVRs with LMMs, incorporating fixed variables including age, sex, education, and APOE genotypes and assessed the association between age and ^18^F-FBP SUVR for each target ROI in each bootstrap sample. We averaged the p-values from all LMM analyses per region and calculated their confidence intervals to evaluate the consistency and detectability of the associations under the three co-registration conditions.

## Results

**Fig. 1:**
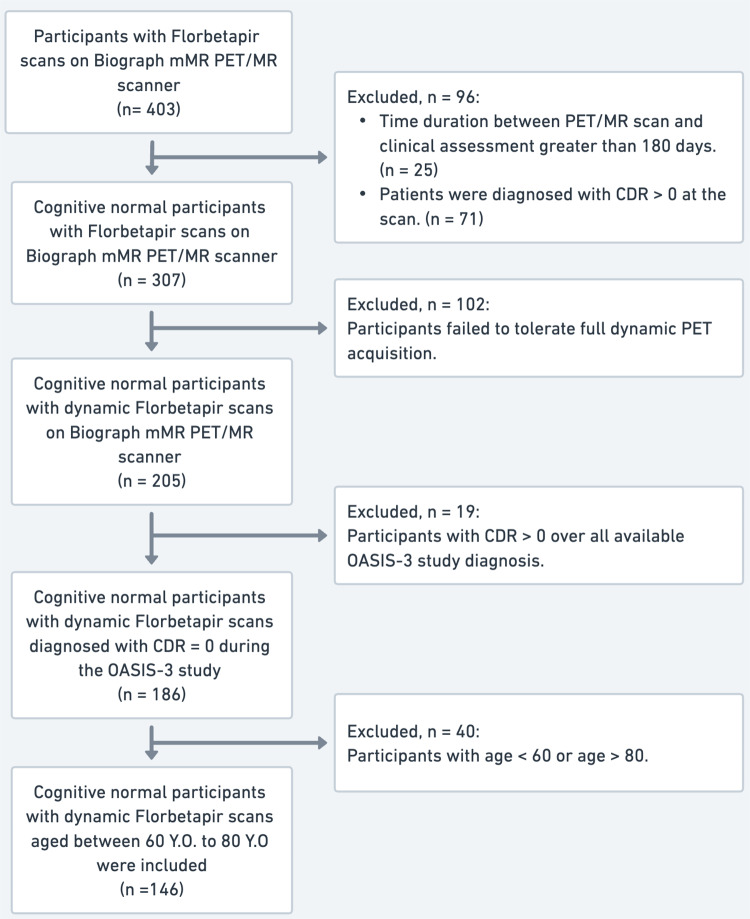
Flow chart of the participants’ selection

After applying the selection criteria, we identified 205 eligible CN participants with dynamic PET/MR scans from the OASIS-3 dataset. 186 participants have CDR of 0 over all available clinical assessments of theirs. 40 participants younger than 60 or older than 80 were excluded. Consequently, a total of 146 participants were included in the final analysis. Each participant had exactly one PET/MR scan available. Figure 1 shows the flowchart for selecting the participants. The demographic characteristics were as follows: the average age was 70.9 ± 4.8 years, with 54.1% of the participants being female. The average years of education were 16.6 ± 2.3 years. 32.2% of participants were APOE ε4 carriers.

### Simulation study

The simulation study was designed to evaluate the performance of TCBC and FSMC in recovering known motion parameters under different noise conditions. Representative axial slices from the PET image with synthetically added noise corresponding to 20-minute and 5-minute acquisitions are shown in Supplementary Figure S1.

**Fig. 2:**
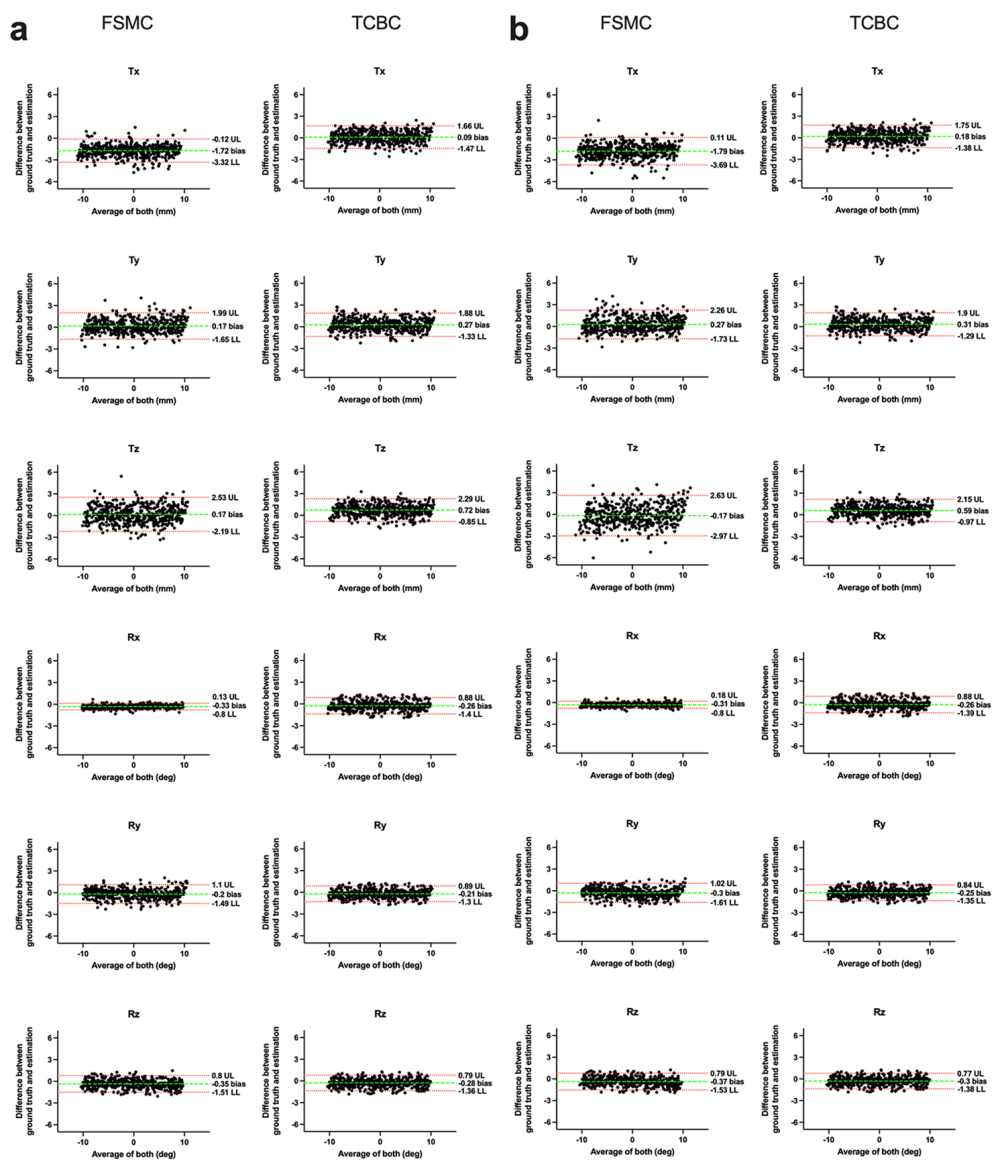
Bland-Altman plots assessing the agreement between ground truth motion parameters and estimations from FSMC and TCBC methods. The analysis presented two simulated noise conditions corresponding to a 20-minute acquisition (Panel **a**) and a 5-minute acquisition (Panel **b**). Each subplot displays the difference between the ground truth and the estimated transformation parameter on the Y-axis to their average on the X-axis. The green dashed central lines represent the mean difference (bias), while the red dotted lines indicate the 95% limits of agreement

The performance metrics for both conditions were summarized in Table 1. Figure 2 illustrates the Bland-Altman plots to assess agreement of each parameter under noise levels of 20-minute (Fig. 2a) and 5-minute (Fig. 2b) acquisitions. For translational parameters, TCBC demonstrated superior performance under both noise conditions. Compared to FSMC, TCBC showed consistent reduction of RMSE by 8.5–58.8%. TCBC also had a higher R-squared values (R²=0.98) in translation across all axes compared to FSMC (R²=0.94–0.97). This enhanced motion correction was also observed from the Bland-Altman plots. The differences for TCBC were more tightly clustered around the mean, yielding narrower limits of agreement than FSMC. Tz was with the greatest difference between TCBC and FSMC. Higher noise led to an increase in RMSE in both methods. For the FSMC method, the translational RMSE increased by 6.8–18.0%. In contrast, the TCBC method increased RMSE by only 5.0% for the X-axis and 1.2% for the Y-axis, while demonstrating a 7.5% decrease for the Z-axis. For rotational parameters, although both methods yielded high consistency, TCBC had a lower RMSE in Ry and Rz but higher RMSE in Rx. The Bland-Altman plots showed that both methods achieved a similar degree of agreement. Speed-wise, TCBC costs approximately 2 to 5 min per scan on workstations with graphical processing units (GPUs) with similar time requirement of FSMC. Overall, these simulation results show that the TCBC method provides a more robust correction for the simulated motion than the MI-based method, and its performance was similar under the two simulated noise levels.

**Table 1:** Performance of co-registration methods under different noise conditions

	*R*-squared						Root mean square error					
Condition Methods	Tx	Ty	Tz	Rx	Ry	Rz	Tx	Ty	Tz	Rx	Ry	Rz
**20-min Noise**												
FSMC	0.97	0.97	0.95	1	0.99	0.99	1.91	0.94	1.22	0.41	0.69	0.69
TCBC	0.98	0.98	0.98	0.99	0.99	0.99	0.80	0.86	1.07	0.64	0.6	0.62
**5-min Noise**												
FSMC	0.97	0.97	0.94	1	0.99	0.99	2.04	1.05	1.44	0.4	0.73	0.7
TCBC	0.98	0.98	0.98	0.99	0.99	0.99	0.84	0.87	0.99	0.63	0.61	0.63

Note. FSMC, FreeSurfer *mri_coreg* co-registration; TCBC: Tracer characteristic-based co-registration

### Detectability of the age-amyloid association

**Fig. 3:**
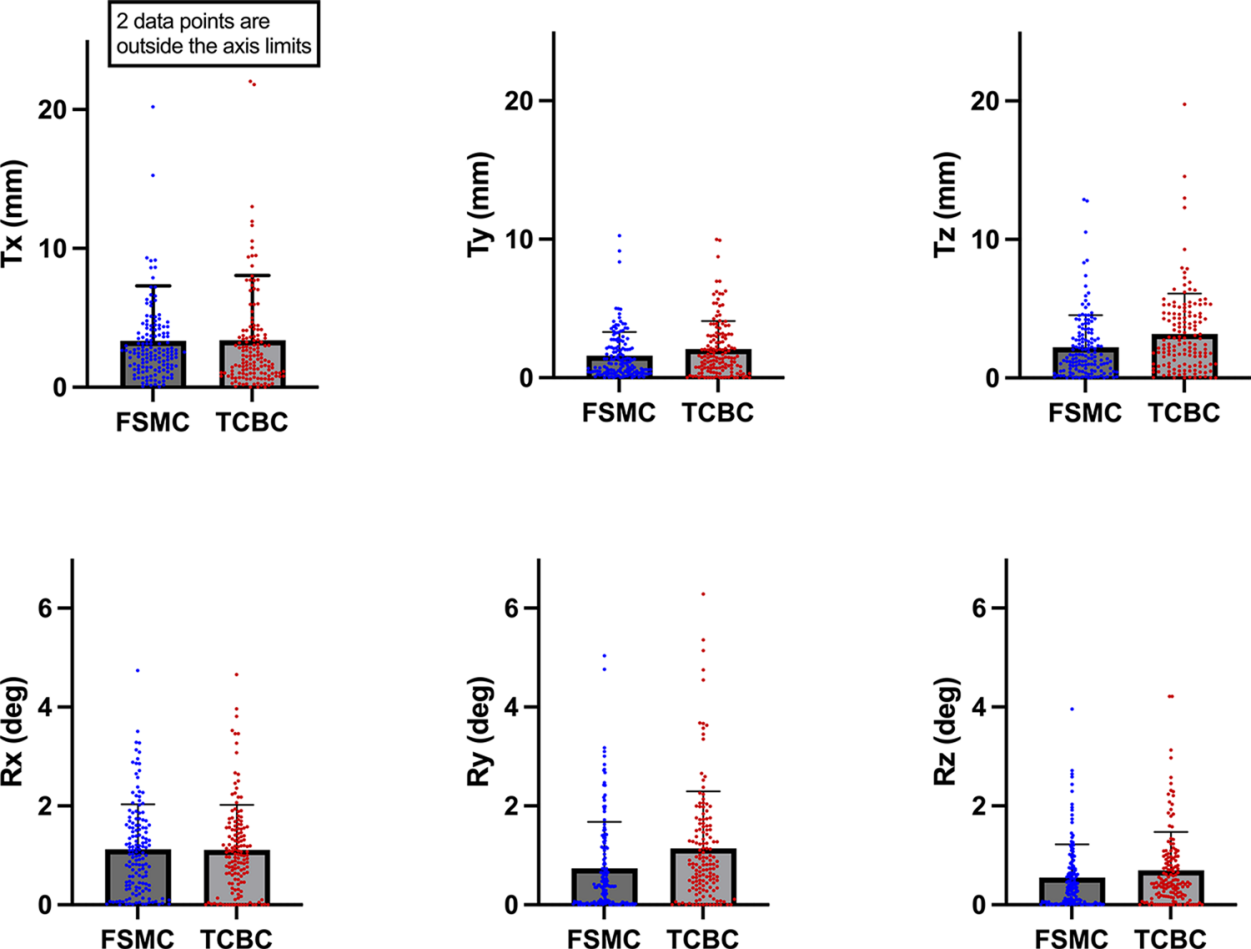
Comparison of translation (Tx, Ty, Tz) and rotation (Rx, Ry, Rz) parameters measured with FSMC and TCBC methods in the complete cohort (*n* = 146). Individual data points are shown to illustrate the range and variability of detected motions within each group. The TCBC method showed an average range of 2.07 mm to 3.38 mm, while FSMC exhibited a comparable range of 1.59 mm to 3.35 mm in translational motions (Tx, Ty, Tz). TCBC detected average rotational motions between 0.74° and 1.14°. FSMC method detected rotational motions between 0.55° to 1.12°. There are extreme cases with translational motions were detected up to approximately 20 mm in the X-axis and 10 mm in the Y- and Z-axes, while rotational motions reached up to 4° to 5° across all axes. Two data points for Tx are outside the axis limits, with translational movements of 38.14 mm detected by FSMC and 40.17 mm detected by TCBC

We first examined the estimated motion with FSMC and TCBC in the complete cohort (*n* = 146). Figure 3 shows the extracted transformation parameters (as absolute values) from both methods across all axes. For translational motions (Tx, Ty, Tz), the mean translation ranged between 2.07 mm and 3.38 mm as measured with TCBC, while FSMC exhibited a comparable range of 1.59 mm to 3.35 mm. For rotational motions (Rx, Ry, Rz), TCBC’s average ranged between 0.74° and 1.14°, whereas FSMC’s average spanned from 0.55° to 1.12°. Certain participants displayed larger motions, with translational movements detected up to approximately 20 mm in X-axis and 10 mm in the Y- and Z-axes, while rotational movements had reached up to 4° to 5°. There was one extreme case with approximately 40 mm motion detected by both methods. Those results suggest that there could still be a significant amount of head motion in simultaneous PET/MR scans and such motion shall be properly addressed through motion correction methods.

Results for the association of age and amyloid burden of the complete cohort and bootstrap samples were summarized in Table 2. In all samples (*n* = 146), uncorrected models revealed significant associations in the precuneus, posterior cingulate, and early Aβ composite regions (*p* < 0.05). After TCBC co-registration, all five target regions showed a significant correlation between age and amyloid burden. On the other hand, FSMC only showed a significant correlation in precuneus, medial orbitofrontal and early Aβ composite regions. In particular, the medial orbitofrontal did not show a significant association under uncorrected data (*p* = 0.378), but a strong association was revealed with FSMC (*p* < 0.01), and an even stronger association was shown under TCBC (*p* < 0.001). As for the bootstrap samples, none of the target ROIs showed a significant association under the uncorrected data. Both TCBC and FSMC methods showed a significant association in the precuneus, medial orbitofrontal, and the early Aβ composite. TCBC yielded the better results in these three regions than FSMC by providing lower means of p-value and tighter confidence intervals in all five target regions than the FSMC-corrected and uncorrected data. Notably, FSMC failed to reduce the means of p-values and narrow the confidence intervals in the anterior cingulate and posterior cingulate regions from uncorrected data, whereas TCBC achieved these reductions.

**Table 2:** Effects of age on the Aβ PET SUVR^a^

	Regions				
	Precuneus	Anterior Cingulate	Posterior Cingulate	Medial Orbitofrontal	Early Aβ Composite
All sample					
Uncorrected	.023*	.053	.021*	.378	.039*
FSMC	.007**	.061	.056	.004**	.006**
TCBC	.004**	.041*	.016*	<.001***	.002**
Bootstrap samples (Mean of p-values [95% CI])					
Uncorrected	.091 [0.085–0.097]	.163 [0.153–0.172]	.096 [0.090–0.103]	.491 [0.475–0.507]	.131 [0.123–0.139]
FSMC	.046* [0.042–0.049]	.172 [0.163–0.183]	.161 [0.151–0.170]	.040* [0.036–0.044]	.045* [0.042–0.049]
TCBC	.032* [0.029–0.035]	.139 [0.130–0.148]	.076 [0.071–0.082]	.008** [0.007–0.010]	.026* [0.023–0.028]

Note. Aβ, amyloid-β; SUVR, standardized uptake value ratio; FSMC, FreeSurfer *mri_coreg* co-registration; TCBC: Tracer characteristic-based co-registration

^a^Unless otherwise indicated, data are expressed as p-value. The effects of age are expressed per 1-U change. All models are adjusted for apolipoprotein E genotype, sex, and education

*p <.05. **p <.01. ***p <.001

**Fig. 4:**
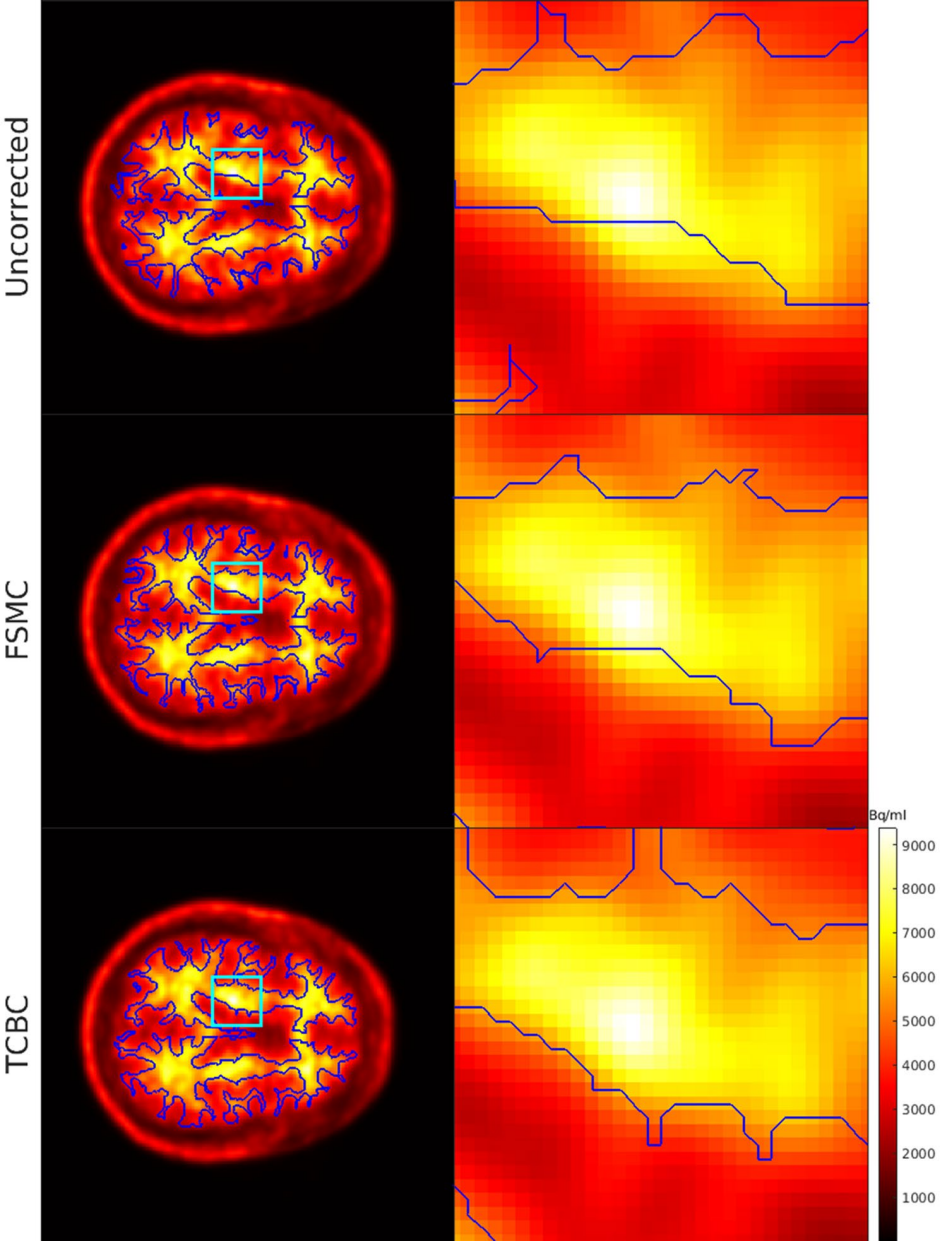
Alignment of the WM contour to the PET images before and after motion correction for a representative subject. WM (blue contour) is derived from FreeSurfer volumetric segmentation. The left column is the same axial slices without correction or after motion correction with FSMC and TCBC methods. The right column is the magnified images from the cyan squares in the left column. The fusion images showed better overlay on the higher ^18^F-FBP uptake pixels after the co-registration

Figure 4 shows an axial PET image of a representative subject from the OASIS-3 dataset fused with the contour of the WM mask before and after motion correction with FSMC or TCBC methods. The magnified images in the right column revealed that both co-registration methods shifted the WM mask towards voxels exhibiting higher ^18^F-FBP uptake and improved the image co-registration.

## Discussion

In this study, we aimed to improve the PET-MR co-registration by proposing the TCBC technique, which incorporated the information of anatomical structures, spatial information, and tracer uptake characteristics to correct the between-modality misalignment in PET/MR images. The motivation for this study was to address the potential and involuntary motion that may still affect the PET-MR co-registration even if the images are acquired from simultaneous PET/MR scanners. The underlying principle of TCBC is to optimize the co-registration by maximizing or minimizing the mean PET signal within a pre-selected anatomical ROI, depending on the tracer uptake characteristics. In the use case of amyloid PET, we utilized the high and uniform non-specific binding in the cerebral WM to guide the optimization. We demonstrated that TCBC provided superior alignment over the MI-based FSMC method in both our simulation and the retrospective human studies. Those data provide initial evidence of the feasibility of using TCBC for PET-to-MR co-registration and motion correction. Considering it is easy to implement and does not require additional data acquisition, such as navigators, TCBC may have good potential for being applied in both clinical and research applications to improve the alignment between PET and MR and to facilitate more precise MR-assisted PET quantification.

Our simulation study was designed to quantitatively compare the accuracy and consistency of TCBC and FSMC under noisy conditions with artificially introduced motion. In general, lower RMSE values were observed in TCBC compared to FSMC, reflecting more accurate recovery of the optimized transformation parameters. Similarly, high R-squared values from linear regression reflected the consistency of TCBC method in estimating the transformation parameters. Noise has affected both methods’ performance, but its impact was less profound in TCBC compared to FSMC. The differences can be attributed to the distinct optimization approaches of the two methods. TCBC focuses on optimizing the mean intensity within the selected ROI as TCBC is guided by the known tracer uptake pattern. In contrast, FSMC optimizes MI between T1w MR and amyloid PET intensities in this study. The signal variability between these two modalities may increase the MI computation variance and cause the optimization moving toward sub-optimal solutions as suggested previously [16]. Overall, our simulation results offered an initial confirmation that TCBC can correct the misalignment between PET and MR and may provide a more consistent and accurate motion correction than MI-based methods.

Even for simultaneous PET/MR imaging, our findings suggest that post-processing motion correction and image re-alignment remains useful and sometimes even essential for improving the PET quantification accuracy. In our retrospective human data analysis, the mean translational motion measured from both FSMC and TCBC ranged from 1.6 to 3.9 mm. Such motion may cause imprecise SUVR measurement, especially in thin structures like the cortex. For example, the mean cortical thickness was reported by Ossenkoppele et al. as 2.2 mm for individuals with preclinical AD and 2.1 mm for those with prodromal AD and AD dementia [40]. The involuntary motion revealed by FSMC and TCBC methods can introduce significant PET-to-MR misalignments as the mean translational motion could be almost twice the cortical thickness. Therefore, a convenient and accurate method for correcting such misalignment is helpful for simultaneous PET/MR imaging when MR-guided PET quantification is desired. TCBC may serve as a useful tool for such image correction as it does not require additional motion tracking equipment [8, 41, 42] or the use of correction algorithms during or before PET data preprocessing or reconstruction [6, 43]. As a post-processing method that makes no adjustment to the image acquisition sequences or protocols, TCBC method can be easily integrated into existing analysis pipelines and performed after completion of a scan. This makes TCBC a practical and potentially useful tool for improving data quality in both retrospective and prospective studies.

We have demonstrated in our study that, by improving the image co-registration with the proposed method, PET quantification may become more precise and help provide a stronger statistical power for an investigational task utilizing PET as the key measurement. From our retrospective human study, we made three interesting findings: Firstly, uncorrected data yielded non-significant or weak associations between age and amyloid deposition in target ROIs, whereas both FSMC and TCBC improved PET measurement precision and enhanced these associations that align with the previous reports [31, 32, 44]. P-values of both the FSMC and TCBC were consistently lower in all five target regions than those of the uncorrected data. Secondly, TCBC further enhanced statistical power in detecting age-related amyloid deposition compared to FSMC, as evidenced by lower p-values across all regions. Thirdly, our bootstrap LMM analyses found that while the uncorrected data consistently failed to show significant associations in all target ROIs, both FSMC and TCBC showed the underlying associations in precuneus, medial orbitofrontal, and early AB composite. Moreover, TCBC demonstrated superior statistical power and consistency, evidenced by the lower mean p-values and narrower confidence intervals when compared to FSMC across all target ROIs. Considering large-scale PET/MR studies could be challenging due to the high cost and technical requirement [45], our proposed method could benefit PET/MR studies with small cohorts by improving the precision of PET quantification and potentially enhancing the statistical power for the desired task of investigation.

Compared to the MI-based methods that consider the volume-wise cross-modality similarities, TCBC does rely on a well-selected target ROI and such dependence must be carefully considered for future applications. In general, an ideal target ROI of TCBC shall meet two criteria. Firstly, it must own a well-characterized tracer uptake pattern with a sufficiently good contrast between the target ROI and the surrounding tissue, so that the optimization algorithm will eventually identify the ideal target ROI location to yield a correct image co-registration. If a disease presents extensive PET abnormalities in the target ROI or large lesions that locally disrupt the image contrast of the target ROI [46, 47], the reduced contrast may mislead the optimization and eventually degrade TCBC’s performance in motion correction. Potential anatomical alternation due to different pathologies shall be considered before applying TCBC under specific tasks. Secondly, the target ROI geometry should preferably be large and complex. A small or symmetric ROI may lead to a ‘pivot error’ that is introduced when the co-registration accurately aligns the ROI itself within the image but poses globally rotational inaccuracy. A large, structurally complex ROI could provide more spatial constraints and help reduce the pivot errors, resulting in a more accurate global alignment. Considering these two criteria, WM may be a suitable choice of target ROI for many brain PET tracers as there is usually a good contrast between it and ventricles, even when the gray-white matter contrast is reduced or insufficient. Furthermore, the signal pattern and intensity distribution of WM typically is uniform and consistent, such as the high and homogeneous non-specific binding in amyloid PET or the low uptake in FDG PET to guide the TCBC algorithm [20, 48]. The complex and edgy contour of WM also makes it less prone to the pivot errors. These properties collectively make WM a good candidate for many tracers. However, users shall carefully evaluate the specific combination of tracer, disease, physiologic conditions and the tasks under investigation for the appropriateness of WM as the target ROI and potentially adjust the selection based on their specific application. There could also be scenarios where there is no proper region to serve as target ROI for TCBC. For such scenarios, other co-registration methods shall be considered over TCBC.

We also wish to point out that this is the first presentation of the proposed TCBC method and there is certainly room for improvement towards the methodology. First, the objective function for TCBC is formulated in this work to optimize a simple and straightforward calculation of mean SUV under a single target ROI. However, the concept of ‘tracer characteristic’ under MR-derived segmentation may be extended beyond a single ROI. If we know a certain tracer has specific and well-known uptake patterns in multiple ROIs, the objective function can be further expanded by considering multiple target ROIs. Moreover, the objective function does not necessarily have to be based on mean SUV. Signal-to-noise ratio or intensity entropy may also be good descriptors of the tracer uptake characteristics. Researchers may even consider adding penalty terms to help achieve better image co-registration, for example, by involving MI in the objective function to achieve a co-registration that balances between tracer uptake and spatial similarities. Second, although we used the Nelder-Mead simplex algorithm implemented as MATLAB’s fminsearch as the optimizer, technically TCBC is not limited to a specific optimizer. The disadvantage of fminsearch is that it is an unconstrained minimization algorithm. More advanced optimization algorithms may further improve TCBC’s performance. For instance, artificial intelligence (AI) methods could be employed as the optimizer to improve optimization efficiency and accuracy. When multiple initial guesses are used for numerical optimization, methods of majority voting may help to decide the optimal solution that the optimizer most frequently converges to and to increase the chance of finding the global minimum [49]. This work is aimed at proposing the TCVC method, formulating it and performing its initial evaluation to demonstrate feasibility. Therefore, we have presented TCBC in a relatively straightforward fashion with a simple objective function and numerical optimization approach, so that the initial validation can be carried out without being over-complicated. With the data presented here, TCBC shall be a promising method that can be further improved, evaluated and applied in the future to enhance the co-registration and quantification accuracy.

Three limitations exist in the TCBC methodology and present study. Firstly, TCBC requires a well-characterized PET uptake pattern and therefore may not be suitable for novel tracers in development. Secondly, there were no truly measured patient motion data from the OASIS study to serve as the ground truth that would have allowed us directly evaluating the TCBC and FSMC performance. Technically, the involuntary motion can be measured by MR navigators or optical tracking system during the image acquisition [8, 42]. However, such measurements are often difficult to make in large-scale human studies. To our best knowledge, OASIS-3 did not perform such measurements. In future work, phantom experiments or PET/MR dataset with measured involuntary motion data can be used to better evaluate and validate TCBC. Lastly, we only used amyloid PET with ^18^F-FBP as an initial evaluation and demonstration of TCBC’s performance. How TCBC performs under other tracers and applications require future studies to investigate.

## Conclusion

We introduced the TCBC method for motion correction and image alignment that utilizes tracer characteristics in a selected target ROI to guide co-registration for simultaneous PET/MR brain scans. Our simulation study demonstrated that TCBC provided more consistent and accurate correction than the MI-based method under noisy conditions. When applied to retrospective human imaging data, it enhanced the detectability of a demonstrative task, showing a good potential for motion correction in both research and clinical studies with simultaneous brain PET/MR.

## Electronic supplementary material

Below is the link to the electronic supplementary material.

Supplementary Material 1

## Data Availability

The datasets used and/or analyzed during the current study are available from the corresponding author on reasonable request.

## Abbreviations

PET: Positron Emission Tomography
MR: Magnetic Resonance
ROI: Region of interest
MI: Mutual information
T1w: T1-weighted
TCBC: Tracer characteristic-based co-registration
FSMC: FreeSurfer *mri_coreg*
OASIS-3: Open Access Series of Imaging Studies-3
SUVR: Standardized uptake value ratio
WM: White matter
GM: Gray matter
CN: Cognitive normal
AD: Alzheimer’s disease
^18^F-FBP: ^18^F-Florbetapir
ADRC: Alzheimer’s Disease Research Center
CDR: Clinical Dementia Rating Scale
Aβ: Amyloid-β
RMSE: Root mean square error
LMM: Linear mixed-effect model
AI: Artificial intelligence
