# Supplementary material for: Motion correction of simultaneous brain PET/MR images based on tracer uptake characteristics
Source: EJNMMI Phys. 2025 Jul 30;12:75. doi: 10.1186/s40658-025-00789-6 (PMC12311070; doi:10.1186/s40658-025-00789-6)
Supplement: Supplementary file 1 — Supplementary Material 1 [file 40658_2025_789_MOESM1_ESM.docx]

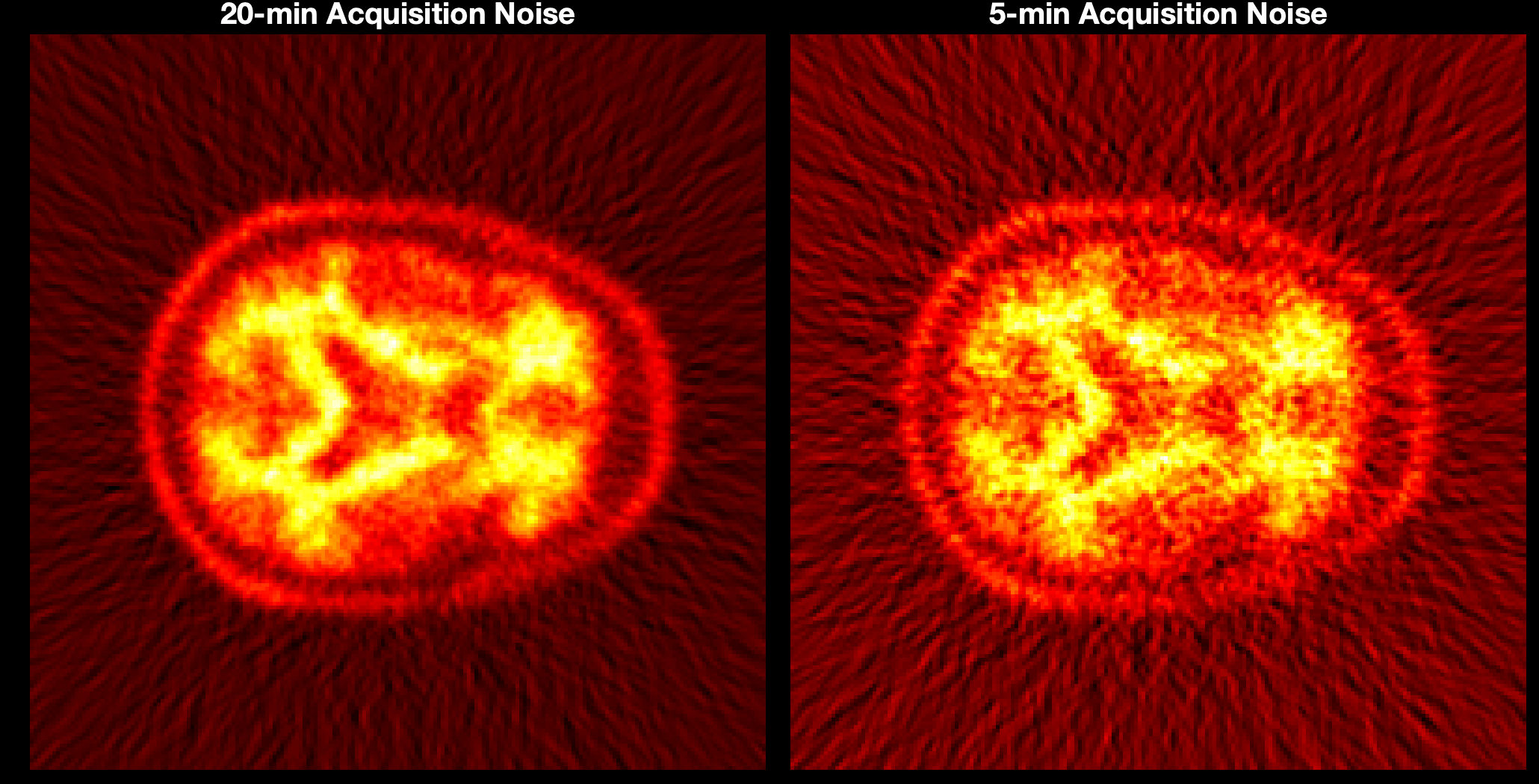


Figure S1. Representative axial slices from the PET image with synthetically added noise corresponding to 20-minute and 5-minute acquisitions.


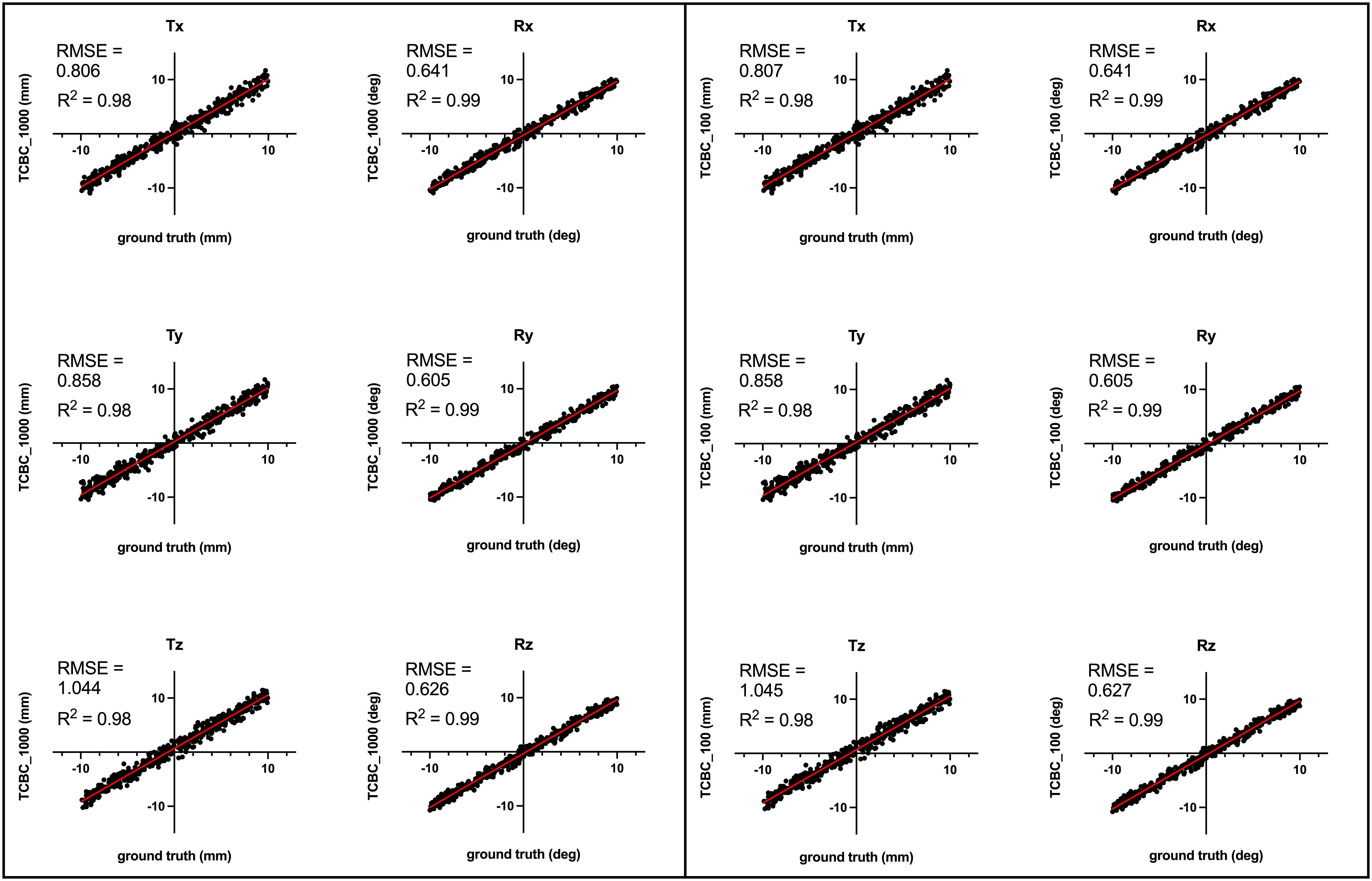


Figure S2. Correlation plots between the estimated co-registration parameters and ground truth parameters with tracer characteristic-based co-registration (TCBC) with 1000-initial-guess (left) and with 100-initial-guess (right)
